# Rooting a phylogenetic tree with nonreversible substitution models

**DOI:** 10.1186/1471-2148-5-2

**Published:** 2005-01-04

**Authors:** Von Bing Yap, Terry Speed

**Affiliations:** 1 Mathematics Department, University of California, 970 Evans Hall, Berkeley, CA 94720, USA; 2 Statistics Department, University of California, 367 Evans Hall, Berkeley, CA 94720, USA; 3 Division of Genetics and Bioinformatics, The Walter & Eliza Hall Institute of Medical Research, 1G Royal Parade, Parkville, Vic 3052, Australia

## Abstract

**Background:**

We compared two methods of rooting a phylogenetic tree: the stationary and the nonstationary substitution processes. These methods do not require an outgroup.

**Methods:**

Given a multiple alignment and an unrooted tree, the maximum likelihood estimates of branch lengths and substitution parameters for each associated rooted tree are found; rooted trees are compared using their likelihood values. Site variation in substitution rates is handled by assigning sites into several classes before the analysis.

**Results:**

In three test datasets where the trees are small and the roots are assumed known, the nonstationary process gets the correct estimate significantly more often, and fits data much better, than the stationary process. Both processes give biologically plausible root placements in a set of nine primate mitochondrial DNA sequences.

**Conclusions:**

The nonstationary process is simple to use and is much better than the stationary process at inferring the root. It could be useful for situations where an outgroup is unavailable.

## Background

Several approaches for inferring a phylogenetic tree from the substitution patterns in multiply aligned sequences are available; they include maximum parsimony, distance-based, maximum likelihood and Bayesian methods [1]. Typically, the inferred tree is unrooted, because the explicit or implicit substitution process used is usually time-reversible. An effective way to put the root on the unrooted tree is to perform a phylogenetic analysis on the sequences of interest together with an outgroup, which is a set of distantly related sequences [2,3]. If the ingroup is monophyletic in the combined phylogenetic tree, then the point where the outgroup touches the ingroup tree is the estimated root. The practical challenge is to find suitable outgroups, and if no such outgroup is available, then one is forced to root the tree using just the ingroup. Several such methods include the molecular clock and nonreversible substitution processes. It seems clear that compared to the outgroup method, the success of these methods is more dependent on the extent to which the accompanying assumptions about the substitution process are satisfied in the data. For example, the molecular clock method should work well if the lineages indeed evolved more or less at the same rate. Likewise, as shown by Huelsenbeck *et al*. [4], a nonreversible process is more likely to succeed the less reversible the real substitution process is.

The nonreversible substitution process, introduced by Yang [5], is *stationary*, i.e., the sequence composition is unchanged in time, and is equal to the equilibrium distribution of the rate matrix *Q*. The consensus is that it does not have enough power to discriminate among the candidate rooted trees. In this paper, we investigate a slightly more general, nonstationary process: in which the initial distribution *π *may not be the equilibrium distribution of the rate matrix *Q*. A priori, giving up stationarity is expected to produce a much better fit to data, since sequence composition is known to evolve, and should be accounted for. Indeed, substitution models where each branch has its own rate matrices had been used to resolve deep splittings in certain phylogenetic trees; see Yang and Roberts, and Galtier and Gouy [6,7]. Our process, which to our knowledge has not been investigated in this context, may be viewed as the simplest case of such nonstationary processes, with many fewer parameters. Thus, it can be used to decide whether the substitution processes on certain branches should be modeled differently. The input to our procedure is a multiple alignment and the topology of an unrooted binary tree. For each rooted tree associated with the given unrooted tree, we seek the maximum likelihood (ML) estimates of the branch lengths, *π *and *Q*. The rooted trees are then ranked in descending order of likelihoods. We model systematic variation in substitution rates among sites by assigning sites into several classes, and the relative rate for each class is estimated by ML; this is equivalent to the combined analysis framework of Yang [8].

We compared the ability of the stationary and nonstationary processes to place the root in three groups of species where the answer is considered well-known: (1) human, chimpanzee and gorilla, (2) human, chimpanzee, gorilla and orangutan, (3) human, mouse, chicken and frog (*xenopus laevis*). The analyses were based on all available mitochondrial protein-coding genes, as well as two nuclear protein-coding genes. Next, we applied the methods to a set of primate mitochondrial DNA sequences.

## Results

### Verification studies

**Figure 1:**
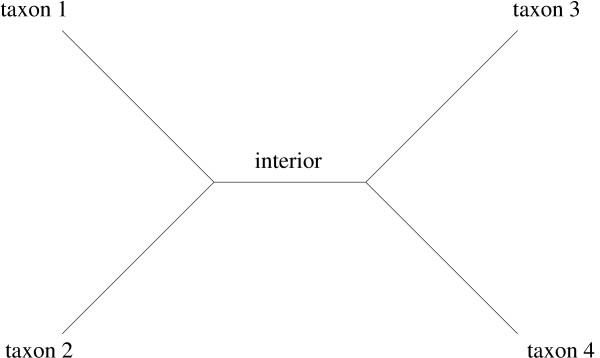
**Unrooted tree with four taxa **The four branches adjacent to leaf nodes will be referred to by the corresponding taxon names.

We fitted the nonstationary (NONSTA), stationary (STA) and reversible (REV) substitution models to all available mitochondrial protein-coding genes, as well as the nuclear genes *albumin *and *c-myc*, for three groups of organisms: (1) human, chimpanzee and gorilla, (2) human, chimpanzee, gorilla and orangutan, and (3) human, mouse, chicken and frog (*xenopus laevis*). The sequences were downloaded from Genbank and aligned using the CLUSTALW alignment of the amino acid sequences. Most alignments looked quite solid [see Additional files]. The beginning of the alignments for the genes *COX1*, *CYTB*, *ND1 *and *ND6 *were slightly adjusted. The root positions are assumed to be on the (1) gorilla, (2) orangutan, and (3) frog branch, respectively. The branches on a tree are referred to by the organism names, except for the case of four taxa, where there is an internal branch (Figure 1). For groups (2) and (3), it was assumed that human was most closely related to chimpanzee and mouse respectively; thus the unrooted tree is determined.

In group 1, the NONSTA and STA processes correctly placed the root in 8 and 6 genes respectively, out of 13 genes (Table 1). In group 2, NONSTA correctly placed the root in 9 genes out of 13 genes, compared to 2 genes for STA (Table 2). In group 3, NONSTA correctly placed the root in 11 genes out of 15 genes, compared to 7 genes for STA (Table 3). Furthermore, NONSTA gives stronger signal, or has better discriminative power: the highest-scoring rooted tree often has noticeably higher log likelihoods than competing rooted trees; this is not so with STA. Thus, NONSTA is much better than STA in placing the root at the individual gene level. Combining the log likelihoods across genes yields overall evidence for the root placements. Table 4 shows that NONSTA is unambiguously correct in all three analyses, while STA only gets the root correctly in group 3, and the signal is weak.

**Table 1:** Human, chimpanzee and gorilla Log-likelihoods (rounded to closest integer) of the MLEs for three rooted trees under the nonstationary (NONSTA), stationary (STA) and reversible (REV) models. If NONSTA or STA places the root correctly, the corresponding log likelihood appears in bold.

gene	root placement	NONSTA	STA	REV
	human	-1320	-1324	-1324
ATP6	chimp	-1318	-1323	-1324
	gorilla	-1318	**-1322**	-1324

	human	-384	-389	-392
ATP8	chimp	-384	-389	-392
	gorilla	-384	-389	-392

	human	-2842	-2876	-2877
COX1	chimp	-2846	-2874	-2876
	gorilla	**-2834**	-2875	-2876

	human	-1285	-1293	-1295
COX2	chimp	-1286	-1294	-1295
	gorilla	**-1281**	**-1292**	-1295

	human	-1477	-1493	-1496
COX3	chimp	-1476	-1493	-1496
	gorilla	**-1472**	-1493	-1496

	human	-2205	-2236	-2236
CYTB	chimp	-2208	-2235	-2236
	gorilla	**-2203**	-2235	-2236

	human	-1787	-1804	-1805
ND1	chimp	-1783	-1804	-1805
	gorilla	**-1776**	**-1802**	-1805

	human	-1949	-1974	-1975
ND2	chimp	-1950	-1974	-1975
	gorilla	**-1941**	-1974	-1975

	human	-663	-679	-680
ND3	chimp	-666	-679	-680
	gorilla	-665	**-679**	-680

	human	-2593	-2612	-2613
ND4	chimp	-2589	-2612	-2613
	gorilla	**-2579**	-2612	-2613

	human	-519	-525	-525.8
ND4L	chimp	-523	-525	-525.8
	gorilla	-520	-526	-525.8

	human	-3600	-3624	-3629
ND5	chimp	-3611	-3628	-3629
	gorilla	**-3583**	**-3628**	-3629

	human	-913	-917	-918
ND6	chimp	-912	-917	-918
	gorilla	-913	**-917**	-918

**Table 2:** Human, chimpanzee, gorilla and orangutan Log-likelihoods (rounded to closest integer) of the MLEs for five rooted trees under the nonstationary (NONSTA), stationary (STA) and reversible (REV) models. If NONSTA or STA places the root correctly, the corresponding log likelihood appears in bold.

gene	root placement	NONSTA	STA	REV
	human	-1649	-1654	-1655
	chimp	-1647	-1654	-1655
ATP6	gorilla	-1647	-1654	-1655
	orangutan	**-1642**	**-1654**	-1655
	interior	-1647	-1654	-1655

	human	-510	-514	-517
	chimp	-510	-515	-517
ATP8	gorilla	-509	-515	-517
	orangutan	-509	-515	-517
	interior	-509	-515	-517

	human	-3456	-3465	-3467
	chimp	-3450	-3464	-3467
COX1	gorilla	-3448	-3465	-3467
	orangutan	**-3437**	-3465	-3467
	interior	-3453	-3465	-3467

	human	-1485	-1496	-1497
	chimp	-1485	-1496	-1497
COX2	gorilla	-1481	-1492	-1497
	orangutan	**-1479**	-1492	-1497
	interior	-1480	-1492	-1497

	human	-1769	-1791	-1796
	chimp	-1780	-1791	-1796
COX3	gorilla	-1781	-1794	-1796
	orangutan	-1772	-1794	-1796
	interior	-1780	-1791	-1796

	human	-2593	-2673	-2674
	chimp	-2594	-2673	-2674
CYTB	gorilla	-2590	-2672	-2674
	orangutan	**-2581**	-2672	-2674
	interior	-2588	-2672	-2674

	human	-2214	-2234	-2236
	chimp	-2210	-2235	-2236
ND1	gorilla	-2205	-2234	-2236
	orangutan	**-2191**	**-2233**	-2236
	interior	-2209	-2235	-2236

	human	-2441	-2469	-2470
	chimp	-2443	-2469	-2470
ND2	gorilla	-2437	-2469	-2470
	orangutan	**-2423**	-2469	-2470
	interior	-2437	-2469	-2470

	human	-837	-855	-856
	chimp	-840	-855	-856
ND3	gorilla	-838	-856	-856
	orangutan	**-834**	-855	-856
	interior	-838	-855	-856

	human	-3151	-3206	-3209
	chimp	-3149	-3205	-3209
ND4	gorilla	-3141	-3205	-3209
	orangutan	-3169	-3207	-3209
	interior	-3145	-3206	-3209

	human	-623	-631	-631
	chimp	-622	-631	-631
ND4L	gorilla	-620	-631	-631
	orangutan	**-619**	-631	-631
	interior	-621	-631	-631

	human	-4469	-4501	-4503
	chimp	-4474	-4502	-4503
ND5	gorilla	-4453	-4502	-4503
	orangutan	**-4448**	-4503	-4503
	interior	-4466	-4502	-4503

	human	-1069	-1076	-1078
	chimp	-1067	-1076	-1078
ND6	gorilla	-1070	-1077	-1078
	orangutan	-1068	-1077	-1078
	interior	-1069	-1076	-1078

**Table 3:** Human, mouse, chicken and frog Log-likelihoods (rounded to closest integer) of the MLEs for five rooted trees under the nonstationary (NONSTA), stationary (STA) and reversible (REV) models. If NONSTA or STA places the root correctly, the corresponding log likelihood appears in bold.

gene	root placement	NONSTA	STA	REV
	human	-7722	-7728	-7731
	mouse	-7708	-7728	-7731
Albumin	chicken	-7723	-7731	-7731
	frog	**-7705**	**-7728**	-7731
	interior	-7723	-7728	-7731

	human	-2608	-2619	-2620
	mouse	-2607	-2619	-2620
ATP6	chicken	-2590	-2619	-2620
	frog	*-2585*	-2618	-2620
	interior	-2585	-2618	-2620

	human	-679	-680	-682
	mouse	-677	-681	-682
ATP8	chicken	-675	-679	-682
	frog	-678	-680	-682
	interior	-675	-680	-682

	human	-3872	-3885	-3887
	mouse	-3869	-3885	-3887
Cmyc	chicken	-3854	-3883	-3887
	frog	**-3814**	**-3882**	-3887
	interior	-3853	-3883	-3887

	human	-4704	-4792	-4794
	mouse	-4709	-4791	-4794
COX1	chicken	-4700	-4794	-4794
	frog	**-4679**	**-4791**	-4794
	interior	-4698	-4792	-4794

	human	-2382	-2399	-2400
	mouse	-2382	-2399	-2400
COX2	chicken	-2377	-2398	-2400
	frog	**-2375**	**-2398**	-2400
	interior	-2376	-2399	-2400

	human	-2502	-2537	-2542
	mouse	-2503	-2540	-2542
COX3	chicken	-2483	-2538	-2542
	frog	-2485	-2539	-2542
	interior	-2486	-2540	-2542

	human	-3782	-3833	-3836
	mouse	-3783	-3832	-3836
CYTB	chicken	-3760	-3832	-3836
	frog	**-3747**	**-3832**	-3836
	interior	-3760	-3833	-3836

	human	-3457	-3483	-3486
	mouse	-3443	-3483	-3486
ND1	chicken	-3435	-3484	-3486
	frog	**-3434**	**-3482**	-3486
	interior	-3442	-3482	-3486

	human	-4275	-4298	-4300
	mouse	-4275	-4298	-4300
ND2	chicken	-4258	-4298	-4300
	frog	**-4253**	**-4296**	-4300
	interior	-4255	-4299	-4300

	human	-1348	-1353	-1355
	mouse	-1347	-1351	-1355
ND3	chicken	-1337	-1353	-1355
	frog	-1335	-1352	-1355
	interior	-1335	-1353	-1355

	human	-5382	-5406	-5406
	mouse	-5380	-5406	-5406
ND4	chicken	-5366	-5404	-5406
	frog	**-5345**	-5405	-5406
	interior	-5365	-5405	-5406

	human	-1259	-1261	-1265
	mouse	-1259	-1264	-1265
ND4L	chicken	-1254	-1262	-1265
	frog	**-1245**	-1263	-1265
	interior	-1254	-1263	-1265

	human	-7053	-7089	-7094
	mouse	-7053	-7091	-7094
ND5	chicken	-7034	-7093	-7094
	frog	**-7006**	-7090	-7094
	interior	-7029	-7091	-7094

	human	-2022	-2025	-2028
	mouse	-2020	-2025	-2028
ND6	chicken	-1995	-2023	-2028
	frog	-1998	-2025	-2028
	interior	-1998	-2025	-2028

**Table 4:** Combined analysis Combined log likelihoods over all genes under the nonstationary (NONSTA), stationary (STA), and reversible (REV) models. If NONSTA or STA places the root correctly, the corresponding log likelihood appears in bold.

group	root placement	NONSTA	STA	REV
	human	-21536	-21743	-21765
1	chimp	-21551	-21746	-21765
	gorilla	**-21470**	-21744	-21765

	human	-26266	-26566	-26589
2	chimp	-26270	-26567	-26589
	gorilla	-26223	-26566	-26589
	orangutan	**-26172**	-26566	-26589
	interior	-26241	-26563	-26589

	human	-53049	-53387	-53427
	mouse	-53029	-53393	-53427
3	chicken	-52848	-53388	-53427
	frog	**-52682**	**-53382**	-53427
	interior	-52833	-53391	-53427

The nuclear genes *albumin *and *c-myc *and three mitochondrial genes, *COX1*, *COX2 *and *ATP6 *from group 3 (with some mouse genes replaced with rat genes) were studied by Huelsenbeck *et al*. [4]. For these five genes, NONSTA and STA performed equally, getting all the correct root placements, except for *ATP6*, with NONSTA again noticeably more discriminative.

### Primate mitochondrial DNA

**Figure 2:**
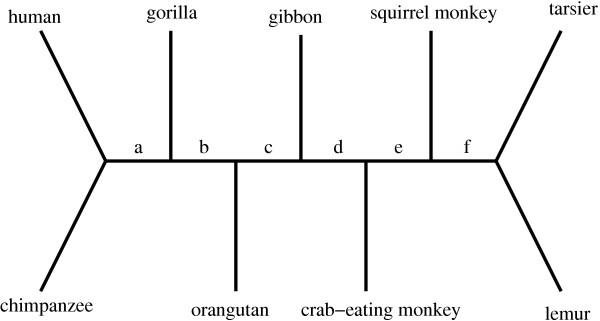
**Unrooted tree for nine primate mtDNA sequences **The assumed unrooted tree is that presented in Yang [5]. The branches adjacent to leaf nodes are referred to by the corresponding organisms, while the interior branches are labelled *a *through *f *as indicated.

Brown *et al*. and Yang [5,9] studied a set of mitochondrial DNA (mtDNA) sequences from human, chimpanzee, gorilla, orangutan, gibbon, crab-eating monkey, squirrel monkey, tarsier and lemur. The topology of Yang's unrooted tree and the branch labels are shown in Figure 2. The mtDNA sequences consist of two protein-coding fragments, separated by three RNA genes. Thus, four site classes are required. Analysis with NONSTA shows that the root is most likely on the tarsier branch, followed closely by the lemur and "f" branches, and the corresponding log likelihoods are quite different from the others (see Table 5). Under STA, the most likely root placements are on the squirrel monkey and lemur branches. Thus, both processes give predictions that are consistent (NONSTA more than STA) with the idea that the root should be somewhere near tarsier and lemur. However, as observed before, NONSTA has much greater discriminative power, and fits the data much better, than STA.

**Table 5:** Nine primates Log-likelihoods (rounded to closest integer) of the MLEs for 15 rooted trees under the nonstationary (NONSTA), stationary (STA) and reversible (REV) models.

root placement	NONSTA	STA	REV
human	-4960	-4965	-4965
chimp	-4959	-4965	-4965
gorilla	-4961	-4965	-4965
orangutan	-4961	-4965	-4965
gibbon	-4962	-4964	-4965
crab-eating macaque	-4955	-4963	-4965
squirrel monkey	-4941	-4961	-4965
tarsier	-4932	-4963	-4965
lemur	-4935	-4961	-4965
a	-4962	-4965	-4965
b	-4961	-4965	-4965
c	-4961	-4964	-4965
d	-4957	-4964	-4965
e	-4948	-4963	-4965
f	-4936	-4963	-4965

## Discussion

Our results confirmed earlier findings that the stationary process (STA) is not very good at discriminating among rooted trees corresponding to the same unrooted tree. In contrast, the nonstationary (NONSTA) process seems much more effective, with individual genes, and with combined genes. It is quite clear that the difference in log likelihoods between fitting STA and the reversible process (REV) is often small, and statistically insignificant, based on the likelihood ratio test, while those between NONSTA and STA, and between NONSTA and REV, are often large, and statistically very significant. Though the chi-square distribution may be inappropriate [10], it seems to be satisfatory in practice [11]. This indicates that NONSTA fits the data much better than STA and REV. Thus it appears that allowing an initial distribution that is uncoupled with the rate matrix gives a better description of the data, and that the greater capacity of NONSTA over STA at estimating the root placement may stem from the ability of NONSTA to allow for some amount of evolution in base composition.

Although Huelsenbeck *et al*.'s analysis using STA failed to place the root correctly in any of the genes *albumin*, *c-myc*, *COX1*, *COX2 *and *ATP6*, there are some differences between the analyses. The raw data were different: the rat *albumin *and *c-myc *genes were used by Huelsenbeck *et al*.; since mouse and rat are very similar, this is not likely to matter much. Secondly, the alignments were probably different, though since the sequences are quite similar, this should not be too important. It is plausible that most of the discrepancies between the results is due to the difference in the estimation procedure (maximum likelihood vs. Bayesian) and to the fact that in Huelsenbeck *et al*., site variation was modeled by the gamma distribution [12], whereas here we only accounted for the codon position effect.

Estimates of the relative rates are quite independent of the model used, and their relative magnitudes are largely within expectations. In particular, for group 3, the relative rates for codon positions 1, 2, and 3 fall between .2 and 1.1, .1 and .6, and 1.5 and 2.7 respectively. For all genes, the third codon position evolved the fastest, followed by the first and second positions. To gauge the contribution from the third codon position, we left out the corresponding bases in group 3 and reran the analysis with NONSTA. This gave the correct root placement in only three genes: *albumin*, *c-myc *and *ND2*, showing the usefulness of the third codon position in this dataset, despite its markedly higher substitution rates. We also found that the pairwise identity at the third codon positions for all genes in groups 3 ranges from 34% to 61%. Base composition being generally nonuniform, the expected pairwise identity at saturation (i.e., infinite evolutionary distance) is lower than 25%. This seems to indicate that the third codon position is not saturated, and hence the phylogenetic information from this position is not just the base composition at each taxon. In addition, the base composition at the third codon position for some genes is quite different from the other positions. Our model does not fit these genes as well as a model where separate processes are associated with the codon positions. Such a model will be investigated in future.

The NONSTA process is only slightly more complicated to apply, compared to the STA and REV processes. The fact that it works quite well in the verification studies and predicts biologically plausible roots for the nine-primate data demonstrates its utility and perhaps argues for its use in routine phylogenetic analysis. In any case, if no suitable outgroup is available, it could be worthwhile to try it. Though the NONSTA process is the most general time-homogeneous Markov process, it is still simplistic and imposes a severe constraint on the evolution of base composition: if two leaf nodes are at the same distance from the root, then the process stipulates that the corresponding sequences must have the same composition. This is patently unrealistic: once lineages split, they should evolve quite independently, and may explain the failure of the process at estimating the root placement for some genes. However, it is still valuable even if it does not always work, in that it can serve as a base from which exploration of richer models can be launched. For instance, one could identify lineages where the evolution significantly deviates from expectations, and then allow these lineages to have different rate matrices, which brings us closer to the very rich models of [6,7,13,14].

## Conclusions

The nonstationary substitution process is simple to use, has much greater power at estimating the root compared to the stationary process, and also fits data much better than the stationary and reversible processes. It seems feasible to use this process in analyses where a suitable outgroup is not easily available. It is also a good starting point for conducting more sophisticated phylogenetic analysis with richer models.

## Methods

Substitutions in DNA sequences are assumed to occur independently at each site according to a Markov process, i.e., given the present base, future substitutions are independent of past substitutions.

Furthermore, it is assumed that the process is time-homogeneous, i.e., substitution rates stay constant in time. As usual, the substitution rate from base *a *to *b *is the (*a*, *b*)-entry in a 4 × 4 rate matrix *Q*; the diagonal entries are such that each row sums to 0. For any *t *> 0, the transition probability *P*(*t*) is given by *P*(*t*) = exp(*Qt*). Let *π *be a probability distribution on the DNA bases. The pair (*π*, *Q*) defines a substitution process on a rooted tree, as follows: pick a base at the root according to *π*, then run the substitution process according to *Q *down the tree, splitting into independent copies whenever a branching is encountered. The joint probability of the observed bases at the leaf nodes can be computed using almost exactly the same algorithm by [15].

There are two important special cases of the time-homogeneous process (*π*, *Q*). Associated with the rate matrix *Q *is a unique distribution *π*_*Q*_, called the equilibrium distribution of *Q*, such that the matrix product *π*_*Q *_× *Q *is the zero vector. The process (*π*_*Q*_, *Q*) is *stationary*, i.e., the sequence composition remains unchanged through time, and is described by *π*_*Q*_. *Q *is said to be *reversible *if it satisfies the detailed balance condition:

Π_*Q*_*Q *= *Q*'Π_*Q*_

where Π_*Q *_is the diagonal form of *π*_*Q *_and *Q*' is the transpose of *Q*. The process (*π*_*Q*_, *Q*) is then *reversible*, i.e., statistically the process looks the same in forward and backward time. In particular, as shown in [15], the joint distribution of the leaf bases is the same regardless of where the root is placed on the tree. The reversible process is known as the REV or time-reversible process in the molecular evolution literature [5,16,17]. Special cases of the REV process include those by Jukes and Cantor, Kimura, Felsenstein (two processes), Hasegawa, Kishino and Yano, and Tamura and Nei [15,18-22]. The nonreversible stationary process was first explored by Yang [5], and subsequently by Huelsenbeck *et al*. [4]. Yang referred to this process as "unrestricted", but we use the abbreviation STA here. We shall refer to the nonstationary process as NONSTA. The numbers of free parameters in the NONSTA, STA and REV processes are respectively 15 (3 in *π *and 12 off-diagonal entries in *Q*), 12 (off-diagonal entries in *Q*) and 9 (3 in *π*_*Q *_and 12 off-diagonal entries in *Q*, minus 6 detailed balance constraints). Since the models are nested, the likelihood ratio test can be used to assess the relative goodness-of-fit of the MLEs. It is standard practice to allow only calibrated rate matrices, i.e., *Q *satisfies



so that a branch length is the average number of substitution events per site. We adopt this practice, and remark that for the nonstationary process (*π*, *Q*), with calibrated *Q*, since in general *π *≠ *π*_*Q*_, it is not true that the expected number of substitutions in 1 time unit is 1, but the difference gets arbitrarily small as time goes to infinity.

The sites in a DNA sequences can have very different substitution rates, the most well-known example being coding sequences, where the third codon positions evolved much faster than the others because of the degeneracy of the genetic code. In cases where the assignment of sites into several classes is known in advance, such as a coding sequence, the easiest way to deal with it is to associate to class *i *an unknown positive number *r*_*i*_, with the constraint that



where *n*_*i *_is the number of sites in class *i*. The relative rate *r*_*i *_either expands or shrinks the tree depending on whether it is more or less than 1. The constraint gives a new interpretation of a branch length: it is now the average over all sites of their expected number of substitutions. Thus, this approach is similar to [8]: effectively, the classes are treated as separate datasets. In this study, coding sequences are divided into three classes by codon position. In the last dataset consisting of nine primate mitochondrial sequences, an additional class is created to account for the RNA-coding bases. Another source of site variation is related to the three-dimensional structure of the protein. For example, hydrophilic residues are usually exposed, hence tend to evolve faster than hydrophobic residues which are deeply buried. Our present approach does not model this and other less obvious sources of site variation. Possible remedies include using the gamma distribution [12] or the hidden Markov model [23].

Given a rooted tree relating aligned coding sequences, we seek the ML estimates of the branch lengths, the substitution parameters, and the relative rates. For other sequences, the relative rates are not estimated. Gradient-based methods are perhaps the most efficient at finding the maximum. The EM algorithm [24] is another possibility. We implemented the simplex method [25], which is slower but is less likely to be misled to local maxima than gradient-based methods. To further reduce the chance of being fooled by local maxima, different initial estimates were used, and the final estimates with the highest likelihood was picked. The initial estimates were obtained by first deriving a reversible rate matrix from a pairwise comparison of two sequences, then using the associated REV process to find the most likely branch lengths and relative rates; all pairwise comparisons were used in this study, so that, for example, four taxa give six initial estimates.

The estimation procedure was implemented in C, and the source code can be requested from the first author.

## Authors' contributions

The idea was conceived by the first author and was inspired and refined by the second author. The first author composed the code and performed the data analysis.

## Supplementary Material

Additional File 1 A text file containing the amino acid sequence alignments for group 1. Click here for file

Additional File 2 A text file containing the amino acid sequence alignments for group 2. Click here for file

Additional File 3 A text file containing the amino acid sequence alignments for group 3. Click here for file
